# Storage conditions for stability of offline measurement of fractional exhaled nitric oxide after collection for epidemiologic research

**DOI:** 10.1186/1471-2466-12-68

**Published:** 2012-11-02

**Authors:** Yoshiko Yoda, Naruhito Otani, Hideki Hasunuma, Hiroshi Kanegae, Masayuki Shima

**Affiliations:** 1 Department of Public Health, Hyogo College of Medicine, 1-1 Mukogawa-cho, Nishinomiya, Hyogo 663-8501, Japan; 2 Center for Environmental Information Science, 8-19 Yonban-cho, Chiyoda-ku, Tokyo, 102-0081, Japan

**Keywords:** Cigarette smoking, Epidemiologic research, Exhaled nitric oxide, Offline measurement, Refrigeration, Storage conditions, Wheezing

## Abstract

**Background:**

The measurement of fractional concentration of nitric oxide in exhaled air (FeNO) is valuable for the assessment of airway inflammation. Offline measurement of FeNO has been used in some epidemiologic studies. However, the time course of the changes in FeNO after collection has not been fully clarified. In this study, the effects of storage conditions on the stability of FeNO measurement in exhaled air after collection for epidemiologic research were examined.

**Methods:**

Exhaled air samples were collected from 48 healthy adults (mean age 43.4 ± 12.1 years) in Mylar bags. FeNO levels in the bags were measured immediately after collection. The bags were then stored at 4°C or room temperature to measure FeNO levels repeatedly for up to 168 hours.

**Results:**

In the bags stored at room temperature after collection, FeNO levels were stable for 9 hours, but increased starting at 24 hours. FeNO levels remained stable for a long time at 4°C, and they were 99.7% ± 7.7% and 101.3% ± 15.0% relative to the baseline values at 24 and 96 hours, respectively. When the samples were stored at 4°C, FeNO levels gradually decreased with time among the subjects with FeNO ≥ 51 ppb immediately after collection, although there were almost no changes among the other subjects. FeNO levels among current smokers increased even at 4°C, although the values among ex-smokers decreased gradually, and those among nonsmokers remained stable. The rate of increase was significantly higher among current smokers than among nonsmokers and ex-smokers from 9 hours after collection onwards.

**Conclusions:**

Storage at 4°C could prolong the stability of FeNO levels after collection. This result suggests that valid measurements can be performed within several days if the samples are stored at 4°C. However, the time course of the changes in FeNO levels differed in relation to initial FeNO values and cigarette smoking.

## Background

Airway inflammation is a central process in various respiratory diseases including asthma [1] and has been directly evaluated by bronchoalveolar lavage and biopsies [2]. However, invasive techniques are needed to obtain these samples [3], and they cannot be used in large-scale epidemiologic research. In addition, eosinophils in sputum, which can be induced using inhalation of hypertonic saline, are used as a marker for airway inflammation [4]. However, the success rates of sputum induction are reported to differ considerably among subjects [5].

The measurement of the fractional concentration of nitric oxide in exhaled air (FeNO) is valuable for the noninvasive and quantitative assessment of airway inflammation [3,6]. Exhaled air can be collected simply and safely [7]. Therefore, FeNO measurement is used widely for the clinical diagnosis and evaluation of respiratory diseases, including asthma [3,8-10]. In addition, the repeated measurement of FeNO is reported to be useful to assess the time course of airway inflammation in asthmatic patients [7,11-13].

For the measurement of FeNO, online and offline methods are available [3]. In the online method, FeNO levels in expired gas are measured directly by a chemiluminescence analyzer [3]. However, only one subject per analyzer can be tested at a time. Because the analyzer is very expensive and not easily portable, the method is unsuitable for large-scale epidemiologic research [14]. In contrast, the offline method, with collection of exhaled air in an aluminized bag for later analysis, has the advantage that it is independent of the analyzer [15]. Because the collection of exhaled air can be performed anywhere and be transported to the laboratory for analysis, this method has been used in some epidemiologic studies [16,17].

Although reasonable agreement has been shown between online and offline measurements [18-20], the measured FeNO values may increase or decrease over time [14,21]. Some investigators found that FeNO levels in the reservoir bag increased after collection [20,22], but others reported that the samples were stable for 24–48 hours [23]. The American Thoracic Society and the European Respiratory Society (ATS/ERS) [3] recommend that FeNO levels of offline samples should be measured within 12 hours from collection. Linn et al. [23] reported that storage under refrigeration can optimize the stability of offline FeNO measurement. Thus, the storage conditions may interfere with the stability of breath samples and should be evaluated in order to standardize the offline method for epidemiologic research. If the storage time after collection can be prolonged, the offline method will become more suitable for field research. In this study, the effects of storage time and temperature on the stability of FeNO measurement in exhaled air after collection were investigated. Furthermore, the requirements for the use of this method in large-scale epidemiologic research were evaluated.

## Methods

### Subjects

The subjects of this study were 48 healthy adults (22 males and 26 females, mean age 43.4 ± 12.1 years). At the beginning of the study, respiratory symptoms and their past history were evaluated by a modified ATS-DLD-78 questionnaire [24]. Wheezing was defined as the occurrence of two or more episodes of chest sounding wheezy or whistling. This study was approved by the Ethics Committee of Hyogo College of Medicine (14-May-2010, approval number 844). The objective and method of this study were fully explained to the subjects, and written, informed consent was obtained from each subject before the study.

### Sampling of exhaled air and measurement of FeNO

Exhaled air samples were collected from the subjects in the morning at their workplace or school. The subjects were requested to refrain from eating, drinking, and smoking for 2 hours before the collection of exhaled air. The collection was performed according to the method described by Saito et al. [18], based on ATS/ERS recommendations [3]. The subjects inspired maximally in the seated position, then exhaled in a 1.5-L Mylar bag (Sievers Instruments, Inc., Boulder, CO, USA) to keep the mouth pressure constant at 15 cmH_2_O and the expiratory flow rate at 50 mL/s. The adjustment was performed with a variable flux pump (Sibata Scientific Technology, Tokyo, Japan), a flow meter (Ono Seisakusho, Tokyo, Japan), and a pressure gauge (Nissin Gauge, Osaka, Japan). The bags were reused up to 20 times and flushed three times with NO-free clean air before reuse. Exhaled air of the first 10 seconds (500 mL) was discarded to prevent contamination by NO from the nasal cavity and upper airway. The collection was repeated six times for each subject. The first measurements of FeNO levels in the bags were performed immediately after collection (within at least 10 min) using a chemiluminescence NO analyzer (Model NA-623N, Kimoto Electric Co, Ltd., Osaka, Japan). The bag samples were then divided into two groups of three samples each and stored at 4°C (refrigeration) and room temperature, respectively. The levels of FeNO in the bags were measured repeatedly at 3, 6, 9, 24, 48, 72, 96, 120, 144, and 168 hours after collection. The analyzer was calibrated daily using zero-gas and standard NO gas (735 ppb). Room temperature during the storage period was 22-28°C, with a mean of 24°C.

### Data analyses

The measured values of FeNO at each time after collection are shown as the means of three samples for each subject at each temperature. If the deviation for each set of triplicate samples was larger than 20% of the mean, the highest FeNO value was removed from the calculation of the mean. Since the FeNO level was roughly log-normally distributed, logarithms of the measurements were used for analysis. The results are expressed as geometric means and 95% confidence intervals (CI). The FeNO levels were compared with respect to sex, age, wheezing, history of allergic rhinitis or pollinosis, and cigarette smoking. The changes in FeNO levels with time after collection were compared by storage temperature. In addition, the subjects were divided into four groups by the quartiles of FeNO levels immediately after collection, and the changes in FeNO levels were evaluated in relation to storage time. Next, FeNO levels at each time point were calculated as percentages relative to that immediately after collection, and they were compared with regard to various factors. All statistical analyses were performed using JMP 9 software (SAS Institute Inc., Cary, NC, USA).

## Results

Table 1 shows the geometric mean FeNO levels by the subjects’ characteristics. There were no significant differences by sex, age group, and history of allergic rhinitis or pollinosis. The geometric mean FeNO level of subjects with wheezing was 57.7 [95% CI, 36.2 to 91.8] ppb, significantly higher than that of subjects without wheezing (30.0 [95% CI, 25.7 to 34.9] ppb). Of the subjects who had wheezing, only one had received medical treatment for asthma. With regard to cigarette smoking, the geometric mean FeNO level in current smokers (22.0 [95% CI, 18.3 to 26.5] ppb) was lower, and that in ex-smokers (41.7 [95% CI, 26.5 to 65.6] ppb) was higher than that in nonsmokers (33.9 [95% CI, 28.1 to 41.0] ppb), although the differences were not significant.

**Table 1:** Geometric mean FeNO levels (ppb) in Mylar bags immediately after collection by subjects’ demographic and health characteristics

	**n**	**Geometric mean**	**(95% confidence interval)**	**p value**
Total	48	33.4	(28.4, 39.3)	
Sex				
Male	22	37.3	(28.8, 48.5)	0.221
Female	26	30.4	(24.9, 37.1)	
Age (years)				
<40	20	31.9	(26.1, 39.0)	0.585
≥40	28	34.6	(27.2, 43.9)	
Wheezing				
Yes	8	57.7	(36.2, 91.8)	0.002
No	40	30.0	(25.7, 34.9)	
Allergic rhinitis or pollinosis				
Yes	20	33.5	(26.3, 42.7)	0.973
No	28	33.3	(27.3, 40.6)	
Cigarette Smoking				
Never	33	33.9	(28.1, 41.0)	0.102
Ever	9	41.7	(26.5, 65.6)	
Current	6	22.0	(18.3, 26.5)	

**Figure 1:**
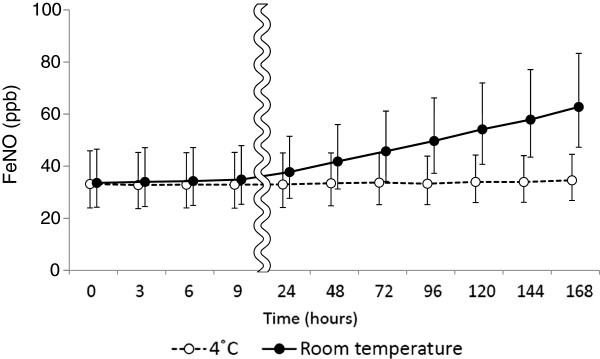
**Changes in FeNO levels (ppb) in Mylar bags with time after collection.** Mylar bags were stored at 4°C (○) and room temperature (·) after collection of exhaled air. All values are geometric means and 95% confidence intervals.

The time courses of the FeNO levels after collection are shown in Figure 1. In the bags stored at room temperature, FeNO levels were nearly stable for 9 hours after collection, but they increased with time in an approximately linear fashion starting at 24 hours. The percentages relative to the baseline values reached 113.5% ± 16.6% and 206.9% ± 102.5% at 24 and 168 hours, respectively. On the other hand, in the bags stored at 4°C, the FeNO levels remained stable for a considerably longer time; they were 99.7% ± 7.7% and 101.3% ± 15.0% relative to the baseline values at 24 and 96 hours, respectively. The subsequent change was also comparatively small, and the value was 106.4% ± 23.5% at 168 hours.

**Figure 2:**
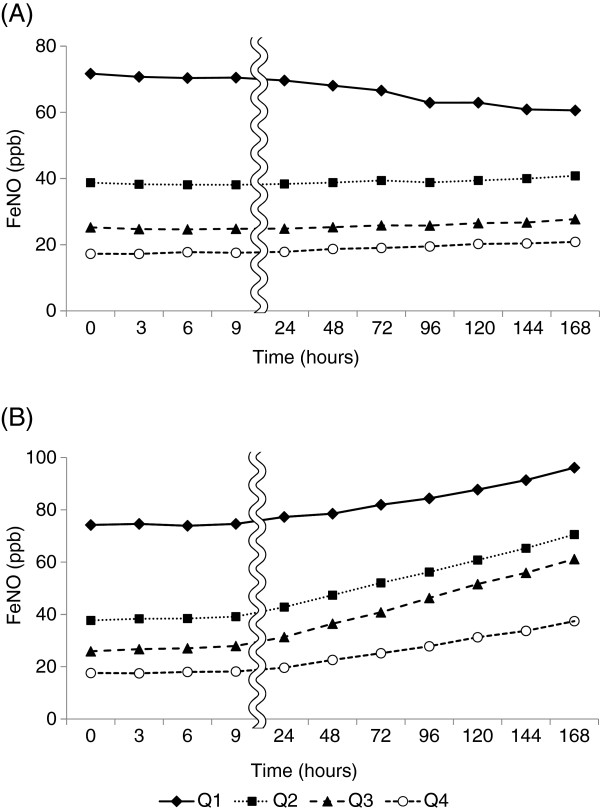
**Changes in FeNO levels (ppb) with time after collection in relation to the initial values.** Mylar bags were stored at 4°C (**A**) and room temperature (**B**) after collection of exhaled air. The subjects were divided into four groups by the quartiles of FeNO levels immediately after collection: Q1, ≥ 51.0 ppb; Q2, 30.4–50.5 ppb; Q3, 22.4–29.0 ppb; Q4, ≤ 20.0 ppb.

When the subjects were divided into four groups by the quartiles of FeNO levels immediately after collection, the time courses of the changes in FeNO levels were examined and are shown in Figure 2. In the bags stored at room temperature, FeNO levels increased with time in all groups, and those in the second and third quartile groups showed marked increases. In contrast, FeNO levels in the bags stored at 4°C gradually decreased with time in the highest quartile group (≥51 ppb immediately after collection). However, there were almost no changes in FeNO levels with time in the other groups.

**Figure 3:**
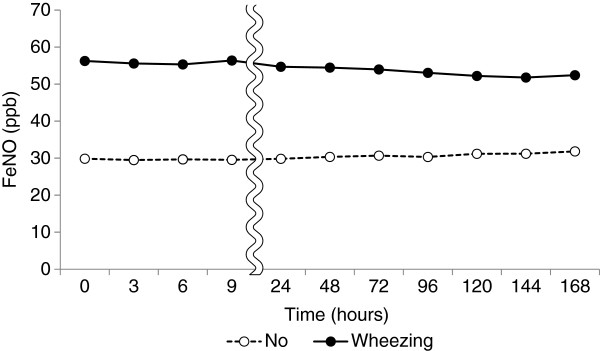
**Changes in FeNO levels (ppb) with time after collection in relation to wheezing.** Mylar bags were stored at 4°C after collection of exhaled air.

**Figure 4:**
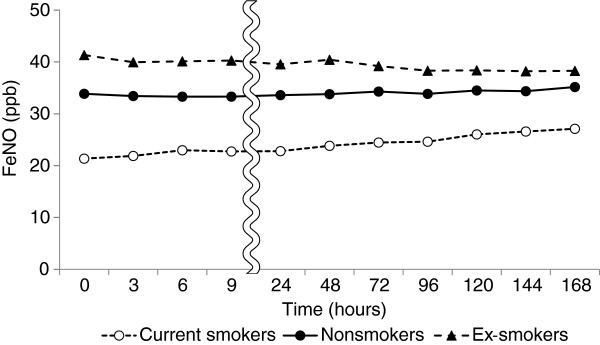
**Changes in FeNO levels (ppb) with time after collection in relation to cigarette smoking.** Mylar bags were stored at 4°C after collection of exhaled air.

The changes in FeNO levels in the bags stored at 4°C relative to the values immediately after collection were compared with regard to various factors (Table 2). There were no significant differences in the changes by sex, age, and history of allergic rhinitis or pollinosis. The FeNO levels decreased slightly with time in the subjects with wheezing and increased in those without (Figure 3); the values were 95.5% ± 22.5% and 108.6% ± 23.4% at 168 hours relative to those immediately after collection, respectively, although the difference was not significant. With regard to cigarette smoking, FeNO levels among current smokers increased with time even in the bags stored at 4°C, and reached 107.4% ± 15.9% and 131.8% ± 43.5% relative to the baseline values at 9 and 168 hours, respectively. On the other hand, the values among ex-smokers decreased gradually with time, and those among nonsmokers remained essentially stable (Figure 4). The rate of increase among current smokers was significantly higher than among nonsmokers and ex-smokers at 9 hours and later.

**Table 2:** Changes in FeNO levels in Mylar bags stored at 4°C after sampling, in relation to various factors

	**n**	**9 hours**			**24 hours**			**72 hours**			**168 hours**		
		**%**	**(SD)**	**p value**	**%**	**(SD)**	**p value**	**%**	**(SD)**	**p value**	**%**	**(SD)**	**p value**
Sex													
Male	22	101.2	(9.0)	0.105	100.3	(10.8)	0.643	101.7	(16.3)	0.799	104.9	(29.8)	0.689
Female	26	97.9	(4.8)		99.2	(3.6)		102.7	(8.5)		107.7	(17.1)	
Age (years)													
<40	20	98.8	(4.0)	0.623	99.3	(4.7)	0.779	101.2	(7.5)	0.646	104.7	(15.0)	0.676
≥40	28	99.8	(8.8)		100.0	(9.4)		103.0	(15.2)		107.6	(28.3)	
Wheezing													
Yes	8	100.3	(4.0)	0.709	97.3	(4.4)	0.340	96.4	(10.0)	0.150	95.5	(22.5)	0.154
No	40	99.2	(7.7)		100.2	(8.2)		103.4	(12.7)		108.6	(23.4)	
Allergic rhinitis or pollinosis													
Yes	20	100.3	(8.7)	0.291	100.3	(9.6)	0.558	103.9	(15.5)	0.292	109.3	(28.9)	0.310
No	28	98.1	(4.1)		98.9	(4.0)		100.0	(6.0)		102.3	(12.4)	
Cigarette Smoking													
Never	33	98.5	(4.5)	0.010	99.3	(3.9)	0.007	101.6	(8.2)	0.003	105.2	(17.1)	0.006
Ever	9	97.5	(2.3)		95.7	(3.0)		95.1	(7.7)		93.9	(15.4)	
Current	6	107.4	(15.9)* ^†^		107.9	(18.4) * ^‡^		116.4	(24.7) * ^‡^		131.8	(43.5) * ^‡^	

Data are shown as percentages relative to the baseline values immediately after sampling.

* p<0.05, significantly different from nonsmokers. ^†^p<0.05; ^‡^p<0.01, significantly different from ex-smokers.

## Discussion

In this study, the effectiveness of offline FeNO measurement after collection was evaluated in terms of its application to epidemiologic research. A number of previous studies reported that FeNO levels were valuable for the quantitative and noninvasive assessment of airway inflammation [3,6,9]. Because the offline method of FeNO measurement has an advantage in that exhaled air can be collected anywhere and from multiple subjects at the same time [20,22], it is considered to be suitable for large-scale field research [25-27]. However, the storage conditions may affect the FeNO levels because of the lag-time from sampling to measurement [14,21]. Therefore, the accuracy of FeNO levels in the offline method should be evaluated after consideration of storage conditions, including temperature and time. The ATS/ERS recommend that offline samples be measured within 12 hours from collection [3]. If the storage time from sampling to measurement can be prolonged, it will be possible to measure FeNO levels for many more people. This raises the possibility that the offline method of FeNO measurement may be used to evaluate the degree of airway inflammation in large-scale epidemiologic research. Previous studies reported that refrigerated storage prolonged the stability of FeNO levels in the bags [21,23,28]. However, most of the previous studies investigated only small numbers of subjects. Few studies have evaluated the time course of the changes in offline FeNO levels among enough subjects including healthy subjects.

The present study demonstrated that FeNO levels remained stable in the bags stored at room temperature for the first 9 hours after collection, but increased starting at 24 hours. When the bags were stored at 4°C, the stability of the FeNO levels was prolonged for a comparatively long time. These findings are consistent with those previously reported by Shimizu et al. [28], who investigated asthma patients. The present study showed that the offline FeNO levels changed in relation to time and temperature among healthy adults, as well as among asthma patients.

Shimizu et al. [28] found that the time course of FeNO levels after collection differed in relation to the initial FeNO levels. In the present study, the time courses of FeNO levels after collection were compared among four groups divided by the quartiles of the initial FeNO levels. In the bags stored at room temperature, FeNO levels increased with time regardless of the initial values. When the bags were stored at 4°C, FeNO levels decreased gradually with time in the group with an initial FeNO level of 51 ppb or above, although the changes in FeNO levels with time were small in the other groups. These findings differ from the results of Shimizu et al. [28] The difference may be due to the fact that almost all of the present subjects were healthy adults.

The subjects with wheezing showed significantly higher FeNO levels immediately after collection than those without, in accordance with many previous studies [18,29,30]. When stored at 4°C, FeNO levels among the subjects with wheezing decreased slightly starting at 24 hours, while the levels among those without wheezing remained stable for a long time. Because the initial FeNO levels among the subjects with wheezing had been quite high, the FeNO levels still remained sufficiently high, even when the levels decreased during storage. Thus, the difference in FeNO levels between the subjects with and without wheezing remained significant. Therefore, it appears that the measurement of FeNO levels after storage at 4°C for several days does not affect the qualitative differentiation of airway inflammation. However, it may be difficult to assess the degree of airway inflammation quantitatively, because FeNO levels decreased gradually among the subjects with wheezing starting at 24 hours. Elevated FeNO levels in subjects with rhinitis have been reported [30-32]. However, in the present study, there were no differences in FeNO levels in relation to a history of allergic rhinitis or pollinosis, though only a history of these diseases, and not present symptoms, was evaluated. The effects of upper airway symptoms, including rhinitis and pollinosis, on FeNO levels have yet to be evaluated.

In this study, FeNO levels immediately after collection were lower among smokers than among non-smokers, despite smoking cessation for 2 hours before the collection of exhaled air. The non-significance of differences in initial FeNO in relation to smoking habits might be due to the small number of current smokers in the study. FeNO levels have been reported to be chronically reduced in cigarette smokers, in addition to the acute effects immediately after smoking [33-35]. The high NO concentration in cigarette smoke may chronically inhibit the activity of NO synthase (NOS) in the respiratory tract [36], resulting in a reduction in FeNO levels among smokers. However, even in the bags stored at 4°C after collection, FeNO levels among smokers started to increase at a relatively early stage and reached 107.4% ± 15.9% relative to the baseline values at 9 hours. On the other hand, FeNO levels among ex-smokers decreased with time, and those among nonsmokers changed very little. Even when the subjects were limited to non-smokers and ex-smokers whose initial FeNO levels were similar to those among current smokers (<30 ppb), the changes in FeNO levels with time were small compared with those among current smokers, although the differences were not significant [see Additional file 1. The mechanisms of these time courses changes in relation to cigarette smoking are unclear. The activity of NOS inhibitors that reduce NO levels in exhaled air of smokers may be diminished during storage. This finding suggests that FeNO levels measured by the offline method may be decreased among smokers even within 12 hours after collection, which is recommended by the ATS/ERS. Various factors, including cigarette smoking, that affect the changes in FeNO levels with time should be further evaluated.

The results of this study should be interpreted cautiously for several reasons. First, the effects of storage conditions other than temperature and time, such as humidity, were not considered. Paredi et al. [37] reported that FeNO levels remained stable for 24 hours in Mylar bags containing silica gel, though controls without silica gel were not investigated. Bondini et al. [21] found that the addition of silica gel increased the variability of FeNO levels during storage. Second, the time courses of FeNO levels in the bags stored at 4°C or room temperature after collection were evaluated; storage at other temperatures was not evaluated. Although the effects of warming or freezing have also been evaluated [21,23], the previous studies showed that FeNO levels in the bags were stable for the longest time under refrigerated storage. In addition, a simple storage condition is desired for epidemiologic research. Third, most of the subjects of this study were healthy subjects, and only one subject had received treatment for asthma. However, eight subjects, including an asthmatic patient, had wheezing, and their FeNO levels were considerably higher than those without wheezing, in agreement with the previous reports [29,30]. Therefore, this method appears to assess airway inflammation adequately. The effect of infections [38] was not evaluated, because none of the subjects had any symptoms associated with respiratory tract infections. Fourth, this study was performed under identical conditions. That is, exhaled air was collected in the morning from the subjects who were requested to refrain from eating, drinking, and smoking for 2 hours before the collection. These conditions should be considered when the offline measurement of FeNO is applied to epidemiologic research.

In summary, this study demonstrated that the stability of FeNO levels after collection was prolonged in the bags stored at 4°C. It is desirable that offline FeNO levels should be measured as soon as possible after collection, as the ATS/ERS recommended measurement within 12 hours. However, the samples collected in field research might not often be measured within this time. The present results suggest that valid measurement can be done within several days after collection if the samples are handled appropriately. However, the time course of the changes in FeNO levels after collection differed in relation to the initial FeNO values and cigarette smoking, not just the storage temperature. Despite these problems, this study shows that the offline method of FeNO measurement can be highly effective for large-scale epidemiologic research under conditions in which the bags including exhaled air are stored at 4°C immediately after collection and are delivered to the laboratory under refrigeration.

## Conclusions

We conclude that valid measurement of FeNO levels can be done within several days after collection if the samples are stored at 4°C. The time course of the changes in FeNO levels after collection differed in relation to the initial FeNO values and cigarette smoking, not just the storage temperature. Despite these problems, this study shows that the offline method of FeNO measurement can be highly effective for large-scale epidemiologic research, under conditions in which the bags including exhaled air are handled appropriately after collection.

## Abbreviations

NO: Nitric oxide; FeNO: Fractional concentration of nitric oxide in exhaled air; ATS: American Thoracic Society; ERS: European Respiratory Society; NOS: Nitric oxide synthase.

## Competing interests

The authors declare that they have no competing interests.

## Authors' contributions

YY, NO, and MS designed the study. YY and NO performed the collection of exhaled air. YY, HH, and HK performed the measurement of FeNO. YY did the statistical analysis and prepared the first draft of manuscript. MS had full access to all the data in the study and takes responsibility for the accuracy of the data analysis. All authors contributed to and approved the final version of the manuscript.

## Pre-publication history

The pre-publication history for this paper can be accessed here:

http://www.biomedcentral.com/1471-2466/12/68/prepub

## Supplementary Material

Additional file 1 **In order to determine whether the different rates of change in NO concentration in the bags stored at 4°C related to smoking category are explained by the differences in initial concentration, the subjects were limited to non-smokers and ex-smokers whose initial FeNO levels were similar to those among current smokers (< 30ppb).** In this case, FeNO levels among non-smokers (n = 14) in the bags stored at 4°C were 98.4% ± 5.3% and 113.8% ± 9.1% relative to the baseline values at 9 and 168 hours, respectively, and the values among ex-smokers (n = 4) were 97.0% ± 2.0% and 105.1% ± 7.4%, respectively. These values were smaller than those among current smokers (n = 6). However, the differences were not significant, because of the small number of subjects. Click here for file

## References

[B1] Shelhamer JH Levine SJ Wu T Jacoby DB Kaliner MA Rennard SI NIH conference. Airway inflammation Ann Intern Med 1995 123 288 304 7611596 10.7326/0003-4819-123-4-199508150-00008

[B2] Foresi A Bertorelli G Pesci A Chetta A Olivieri D Inflammatory markers in bronchoalveolar lavage and in bronchial biopsy in asthma during remission Chest 1990 98 528 535 10.1378/chest.98.3.528 2203613

[B3] American Thoracic S, European Respiratory S ATS/ERS recommendations for standardized procedures for the online and offline measurement of exhaled lower respiratory nitric oxide and nasal nitric oxide, 2005 Am J Respir Crit Care Med 2005 171 912 930 15817806 10.1164/rccm.200406-710ST

[B4] Pin I Gibson PG Kolendowicz R Girgis-Gabardo A Denburg JA Hargreave FE Dolovich J Use of induced sputum cell counts to investigate airway inflammation in asthma Thorax 1992 47 25 29 10.1136/thx.47.1.25 1539140 PMC463545

[B5] Gibson PG Henry RL Thomas P Noninvasive assessment of airway inflammation in children: induced sputum, exhaled nitric oxide, and breath condensate Eur Respir J 2000 16 1008 1015 11153569

[B6] Barnes PJ Belvisi MG Nitric oxide and lung disease Thorax 1993 48 1034 1043 10.1136/thx.48.10.1034 7903007 PMC464825

[B7] Dweik RA Boggs PB Erzurum SC Irvin CG Leigh MW Lundberg JO Olin AC Plummer AL Taylor DR American Thoracic Society Committee on Interpretation of Exhaled Nitric Oxide Levels for Clinical A An official ATS clinical practice guideline: interpretation of exhaled nitric oxide levels (FENO) for clinical applications Am J Respir Crit Care Med 2011 184 602 615 10.1164/rccm.9120-11ST 21885636 PMC4408724

[B8] Lundberg JO Weitzberg E Lundberg JM Alving K Nitric oxide in exhaled air Eur Respir J 1996 9 2671 2680 10.1183/09031936.96.09122671 8980984

[B9] Kharitonov SA Barnes PJ Exhaled markers of pulmonary disease Am J Respir Crit Care Med 2001 163 1693 1722 11401895 10.1164/ajrccm.163.7.2009041

[B10] Smith AD Cowan JO Filsell S McLachlan C Monti-Sheehan G Jackson P Taylor DR Diagnosing asthma: comparisons between exhaled nitric oxide measurements and conventional tests Am J Respir Crit Care Med 2004 169 473 478 14644933 10.1164/rccm.200310-1376OC

[B11] Matsunaga K Yanagisawa S Hirano T Ichikawa T Koarai A Akamatsu K Sugiura H Minakata Y Matsunaga K Kawayama T Ichinose M Associated demographics of persistent exhaled nitric oxide elevation in treated asthmatics Clin Exp Allergy 2012 42 775 781 10.1111/j.1365-2222.2011.03945.x 22515393

[B12] Kharitonov SA Barnes PJ Does exhaled nitric oxide reflect asthma control? Yes, it does! Am J Respir Crit Care Med 2001 164 727 728 11549522 10.1164/ajrccm.164.5.2106122c

[B13] Strunk RC Szefler SJ Phillips BR Zeiger RS Chinchilli VM Larsen G Hodgdon K Morgan W Sorkness CA Lemanske RF Jr Relationship of exhaled nitric oxide to clinical and inflammatory markers of persistent asthma in children J Allergy Clin Immunol 2003 112 883 892 10.1016/j.jaci.2003.08.014 14610474

[B14] Jöbsis Q Schellekens SL Kroesbergen A Hop WC de Jongste JC Off-line sampling of exhaled air for nitric oxide measurement in children: methodological aspects Eur Respir J 2001 17 898 903 10.1183/09031936.01.17508980 11488323

[B15] Jöbsis Q Schellekens SL Kroesbergen A Hop WC de Jongste JC Sampling of exhaled nitric oxide in children: end-expiratory plateau, balloon and tidal breathing methods compared Eur Respir J 1999 13 1406 1410 10.1183/09031936.99.13614119 10445620

[B16] Eckel SP Berhane K Salam MT Rappaport EB Linn WS Bastain TM Zhang Y Lurmann F Avol EL Gilliland FD Residential traffic-related pollution exposures and exhaled nitric oxide in the children’s health study Environ Health Perspect 2011 119 1472 1477 10.1289/ehp.1103516 21708511 PMC3230449

[B17] Bastain TM Islam T Berhane KT McConnell RS Rappaport EB Salam MT Linn WS Avol EL Zhang Y Gilliland FD Exhaled nitric oxide, susceptibility and new-onset asthma in the Children’s Health Study Eur Respir J 2011 37 523 531 10.1183/09031936.00021210 20634264 PMC4020940

[B18] Saito J Sato S Hasunuma H Ishimaru Y Kanegae H Kudo S Munakata M Off-line fractional exhaled nitric oxide measurement is useful to screen allergic airway inflammation in an adult population J Asthma 2007 44 805 810 10.1080/02770900701645595 18097854

[B19] Linn WS Berhane KT Rappaport EB Bastain TM Avol EL Gilliland FD Relationships of online exhaled, offline exhaled, and ambient nitric oxide in an epidemiologic survey of schoolchildren J Expo Sci Environ Epidemiol 2009 19 674 681 10.1038/jes.2008.64 18941479

[B20] Silkoff PE Stevens A Pak J Bucher-Bartelson B Martin RJ A method for the standardized offline collection of exhaled nitric oxide Chest 1999 116 754 759 10.1378/chest.116.3.754 10492283

[B21] Bodini A Pijnenburg MW Boner AL de Jongste JC Exhaled nitric oxide in mylar balloons: influence of storage time, humidity and temperature Mediators Inflamm 2003 12 47 49 10.1080/0962935031000096971 12745548 PMC1781591

[B22] Barreto M Villa MP Martella S Falasca C Guglielmi F Pagani J Darder MT Ronchetti R Off-line exhaled nitric oxide measurements in children Pediatr Pulmonol 2001 32 159 167 11477733 10.1002/ppul.1102

[B23] Linn WS Avila M Gong H Jr Exhaled nitric oxide: sources of error in offline measurement Arch Environ Health 2004 59 385 391 10.3200/AEOH.59.8.385-391 16268114 PMC1866170

[B24] Ferris BG Epidemiology Standardization Project (American Thoracic Society) Am Rev Respir Dis 1978 118 1 120 742764

[B25] Linn WS Rappaport EB Berhane KT Bastain TM Avol EL Gilliland FD Exhaled nitric oxide in a population-based study of southern California schoolchildren Respir Res 2009 10 28 10.1186/1465-9921-10-28 19379527 PMC2678086

[B26] Spanier AJ Hornung RW Kahn RS Lierl MB Lanphear BP Seasonal variation and environmental predictors of exhaled nitric oxide in children with asthma Pediatr Pulmonol 2008 43 576 583 10.1002/ppul.20816 18429012 PMC3483596

[B27] Perzanowski MS Divjan A Mellins RB Canfield SM Rosa MJ Chew GL Rundle A Goldstein IF Jacobson JS Exhaled NO among inner-city children in New York City J Asthma 2010 47 1015 1021 10.3109/02770903.2010.513075 20936992 PMC3056403

[B28] Shimizu H Obase Y Ikeda M Kurose K Abe M Mouri K Katoh S Miyashita N Kobashi Y Oka M Stability of sealed-bag samples for off-line measurement of fractional exhaled nitric oxide Ann Allergy Asthma Immunol 2011 106 378 380 10.1016/j.anai.2011.01.010 21530868

[B29] Baraldi E Dario C Ongaro R Scollo M Azzolin NM Panza N Paganini N Zacchello F Exhaled nitric oxide concentrations during treatment of wheezing exacerbation in infants and young children Am J Respir Crit Care Med 1999 159 1284 1288 10194178 10.1164/ajrccm.159.4.9807084

[B30] Nordvall SL Janson C Kalm-Stephens P Foucard T Toren K Alving K Exhaled nitric oxide in a population-based study of asthma and allergy in schoolchildren Allergy 2005 60 469 475 10.1111/j.1398-9995.2005.00735.x 15727578

[B31] Malinovschi A Alving K Kalm-Stephens P Janson C Nordvall L Increased exhaled nitric oxide predicts new-onset rhinitis and persistent rhinitis in adolescents without allergic symptoms Clin Exp Allergy 2012 42 433 440 10.1111/j.1365-2222.2011.03947.x 22356144

[B32] Martin U Bryden K Devoy M Howarth P Increased levels of exhaled nitric oxide during nasal and oral breathing in subjects with seasonal rhinitis J Allergy Clin Immunol 1996 97 768 772 10.1016/S0091-6749(96)80154-0 8613633

[B33] Robbins RA Millatmal T Lassi K Rennard S Daughton D Smoking cessation is associated with an increase in exhaled nitric oxide Chest 1997 112 313 318 10.1378/chest.112.2.313 9266863

[B34] Kharitonov SA Robbins RA Yates D Keatings V Barnes PJ Acute and chronic effects of cigarette smoking on exhaled nitric oxide Am J Respir Crit Care Med 1995 152 609 612 7543345 10.1164/ajrccm.152.2.7543345

[B35] Schilling J Holzer P Guggenbach M Gyurech D Marathia K Geroulanos S Reduced endogenous nitric oxide in the exhaled air of smokers and hypertensives Eur Respir J 1994 7 467 471 10.1183/09031936.94.07030467 8013603

[B36] Horvath I Donnelly LE Kiss A Balint B Kharitonov SA Barnes PJ Exhaled nitric oxide and hydrogen peroxide concentrations in asthmatic smokers Respiration 2004 71 463 468 10.1159/000080630 15467323

[B37] Paredi P Loukides S Ward S Cramer D Spicer M Kharitonov SA Barnes PJ Exhalation flow and pressure-controlled reservoir collection of exhaled nitric oxide for remote and delayed analysis Thorax 1998 53 775 779 10.1136/thx.53.9.775 10319060 PMC1745326

[B38] Kharitonov SA Yates D Barnes PJ Increased nitric oxide in exhaled air of normal human subjects with upper respiratory tract infections Eur Respir J 1995 8 295 297 10.1183/09031936.95.08020295 7538934

