# Supplementary material for: Storage conditions for stability of offline measurement of fractional exhaled nitric oxide after collection for epidemiologic research
Source: BMC Pulm Med. 2012 Nov 2;12:68. doi: 10.1186/1471-2466-12-68 (PMC3515473; doi:10.1186/1471-2466-12-68)
Supplement: Additional file 1 — In order to determine whether the different rates of change in NO concentration in the bags stored at 4°C related to smoking category are explained by the differences in initial concentration, the subjects were limited to non-smokers and ex-smokers whose initial FeNO levels were similar to those among current smokers (< 30ppb). In this case, FeNO levels among non-smokers (n = 14) in the bags stored at 4°C were 98.4% ± 5.3% and 113.8% ± 9.1% relative to the baseline values at 9 and 168 hours, respectively, and the values among ex-smokers (n = 4) were 97.0% ± 2.0% and 105.1% ± 7.4%, respectively. These values were smaller than those among current smokers (n = 6). However, the differences were not significant, because of the small number of subjects. [file 1471-2466-12-68-S1.doc]

Additional file 1

In order to determine whether the different rates of change in NO concentration in the bags stored at 4°C related to smoking category are explained by the differences in initial concentration, the subjects were limited to non-smokers and ex-smokers whose initial FeNO levels were similar to those among current smokers (< 30ppb). In this case, FeNO levels among non-smokers (n = 14) in the bags stored at 4°C were 98.4% ± 5.3% and 113.8% ± 9.1% relative to the baseline values at 9 and 168 hours, respectively, and the values among ex-smokers (n = 4) were 97.0% ± 2.0% and 105.1% ± 7.4%, respectively. These values were smaller than those among current smokers (n = 6). However, the differences were not significant, because of the small number of subjects.

Table. Changes in FeNO levels in Mylar bags stored at 4°C after sampling　by cigarette smoking status.

|  |  | 9 hours | | |  | 24 hours | | |  | 72 hours | | |  | 168 hours | | |
| --- | --- | --- | --- | --- | --- | --- | --- | --- | --- | --- | --- | --- | --- | --- | --- | --- |
|  | n | % | (SD) | p value |  | % | (SD) | p value |  | % | (SD) | p value |  | % | (SD) | p value |
| Never | 14 | 98.4 | (5.3) | 0.102 |  | 100.1 | (4.2) | 0.128 |  | 105.2 | (7.1) | 0.126 |  | 113.8 | (9.1) | 0.161 |
| Ever | 4 | 97.0 | (2.0) |  |  | 95.4 | (3.1) |  |  | 98.8 | (7.5) |  |  | 105.1 | (7.4) |  |
| Current | 6 | 107.4 | (15.9) |  |  | 107.9 | (18.4) |  |  | 116.4 | (24.7) |  |  | 131.8 | (43.5) |  |

Data are shown as percentages relative to the baseline values immediately after sampling.

**Figure. Changes in FeNO levels (ppb) with time after collection by cigarette smoking status.**

Mylar bags were stored at 4°C after collection of exhaled air.
